# Social Inequalities in Environmental Resources of Green and Blue Spaces: A Review of Evidence in the WHO European Region

**DOI:** 10.3390/ijerph16071216

**Published:** 2019-04-04

**Authors:** Steffen Andreas Schüle, Lisa Karla Hilz, Stefanie Dreger, Gabriele Bolte

**Affiliations:** 1 Institute of Public Health and Nursing Research, Department of Social Epidemiology, University of Bremen 28359 Bremen, Germany; lisa-hilz@uni-bremen.de (L.K.H.); stefanie.dreger@uni-bremen.de (S.D.); gabriele.bolte@uni-bremen.de (G.B.); 2 Health Sciences Bremen, University of Bremen, 28359 Bremen, Germany

**Keywords:** environmental inequalities, environmental justice, systematic review, green space, blue space, Europe

## Abstract

Residential green and blue spaces and their potential health benefits have received increasing attention in the context of environmental health inequalities, because an unequal social distribution of these resources may contribute to inequalities in health outcomes. This systematic review synthesised evidence of environmental inequalities, focusing on availability and accessibility measures of green and blue spaces. Studies in the World Health Organisation (WHO) European Region published between 2010 and 2017 were considered for the review. In total, 14 studies were identified, where most of them (*n* = 12) analysed inequalities of green spaces. The majority had an ecological study design that mostly applied deprivation indices on the small area level, whereas cross-sectional studies on the individual level mostly applied single social measures. Ecological studies consistently showed that deprived areas had lower green space availability than more affluent areas, whereas mixed associations were found for single social dimensions in cross-sectional studies on the individual level. In order to gain more insights into how various social dimensions are linked to the distribution of environmental resources within the WHO European Region, more studies are needed that apply comparable methods and study designs for analysing social inequalities in environmental resources.

## 1. Introduction

There is increasing attention in Europe to what extent social inequalities in health are related to unequal distributions of environmental burdens and resources, which are described as environmental health inequalities [1]. Many conceptual models have been developed in order to capture the multidimensional relationships between socioeconomic position (SEP), environmental exposures and health on various levels, for example on the small area or individual level [2]. According to Krieger et al., SEP combines actual economic and social resources with prestige-based characteristics that relatively position individuals, households or neighbourhoods in society [3,4]. This review refers to this concept of SEP comprising various socioeconomic characteristics with the additional consideration of further relevant social dimensions, such as gender, ethnicity or age. Furthermore, the term ‘inequality’ is used in this review because the focus is on observable and measurable differences in environmental resources across social groups. The term ‘inequity’ additionally integrates normative aspects assessing which differences are judged as unfair and unjust and therefore need appropriate actions [5].

Most conceptual models of environmental health inequalities share two important hypotheses that originate from the environmental justice debate in the United States and environmental epidemiologic evidence. The first hypothesis suggests that socially deprived areas or individuals are exposed to higher environmental burdens and to a lower availability of environmental resources than more affluent areas or individuals [6,7,8,9]. As a result, adverse environmental health effects would be more prevalent in low SEP groups than in high SEP groups. The second hypothesis assumes that, given the same magnitude of an environmental exposure, adverse environmental health effects are stronger in low SEP groups due to an increased vulnerability [10]. Both pathways may contribute individually or in combination to environmentally driven health disparities. Therefore, there is a need for systematic monitoring of environmental inequalities [1].

Distributional justice, procedural justice, and recognition have been discussed as significant components of environmental justice [11,12]. This review focused on distributional justice in terms of equal access to green or blue spaces across all social groups. Evidence from Europe suggests that population groups with a low SEP have a higher exposure towards environmental burdens related to housing conditions or noise and air pollution [1,13,14,15]. On the side of environmental resources, green spaces and their potential health benefits have been increasingly investigated for the last 10 years and evidence suggests that green spaces are associated with better health and health-promoting behaviours [16,17,18]. Often, there is no clear differentiation from blue spaces as a second environmental resource because studies combine green spaces with elements of blue space, such as beaches [19,20]. However, reviews exclusively focusing on blue spaces elucidate their health-promoting role [21,22].

Assuming that environmental resources are health-promoting, a socioeconomic or sociodemographic unequal distribution would result in an unequal distribution of positive health outcomes related to green or blue space exposure. Therefore, green spaces as an environmental resource have received increasing attention in the context of environmental inequalities [23,24,25,26]. Reviews on inequalities of green space access showed that heterogeneous measures of green space access were used, probably resulting in inconclusive findings on inequalities [23,25,26]. A systematic review from Rigolon on urban park inequalities in mainly industrialised countries found that for proximity measures contrasting relations exist, which means that low SEP groups had both shorter and longer distances to parks depending on the study. For measures of park area or park quality, evidence was more conclusive, pointing at a lower availability and quality of parks for low SEP groups [25]. A second review from Rigolon et al. focusing on inequalities of green spaces in cities of the Global South found more conclusive results for the three measures of proximity, quantity and quality, which means higher distances to green spaces and lower availability or quality of green spaces for low SEP groups [26].

There is a request to integrate perspectives of environmental inequalities and environmental justice into green space management, planning and research in European cities, as there is little knowledge how urban green space availability, accessibility and quality are related to the increasing socioeconomic disparities in European cities [27]. Systematic evidence in Europe is still lacking concerning which methods of green space assessment are applied and which SEP indicators are used. Moreover, there is a lack of systematic knowledge on the extent to which SEP indicators, green space measures and regional differences may influence potential relationships between SEP and the distribution of resources. Therefore, the objective of this systematic review was to synthesise the evidence base on environmental inequalities in the World Health Organisation (WHO) European Region, focusing on availability of or access to green and blue space.

## 2. Materials and Methods

This systematic review has been registered with the PROSPERO international prospective register of systematic review database (Registration number: CRD42018099460) and was carried out following the PRISMA statement [28].

We defined socioeconomic and sociodemographic factors according to the PROGRESS-Plus framework proposed by the Campbell and Cochrane Equity Methods Group [29], which represents eight dimensions across which inequalities may exist. PROGRESS stands for: place of residence, race/ethnicity/culture/language, occupation, gender/sex, religion, education, socioeconomic status and social capital [30]. ‘Plus’ considers other characteristics of populations that may be associated with social disadvantage (e.g., age or disability) [31]. We systematically analysed the study results according to the hypothesis that socially disadvantaged individuals or areas have less environmental resources. In the case of the social dimensions age and gender, old people, compared to other age groups, and females, compared to males, were regarded as socially disadvantaged groups.

We defined environmental resources as measures of green or blue space either objectively or subjectively assessed. Proxy measures for green and blue space were also considered, such as vegetation indices or measures of planting.

### 2.1. Search Strategy

The three electronic databases MEDLINE (via PubMed), Scopus and Web of Science were searched on 23 April 2018. Search terms included keywords for green and blue space combined with terms for sociodemographic and socioeconomic characteristics and with terms for inequality, inequity or environmental justice using Boolean operators (see Table 1 and Supplementary Materials S3). In PubMed, terms of Medical Subject Headings were additionally considered (see Table 1).

Evidence obtained with this review will be part of an update of the report ‘Environmental Health Inequalities in Europe’, which was published by the WHO Regional Office for Europe in 2012 [1]. Therefore, search results were restricted to articles published between 1 January 2010 and 31 December 2017.

### 2.2. Eligibility Criteria for Title, Abstract and Full-Text Screening

We included only observational studies (cohort, cross-sectional or ecological) conducted in the 53 Member States belonging to the WHO European Region [32] and published in peer-reviewed journals in the English language. Studies had to be quantitative; qualitative studies as well as studies with a research focus on animals and their environments were excluded.

### 2.3. Data Collection and Synthesis

**Figure 1:**
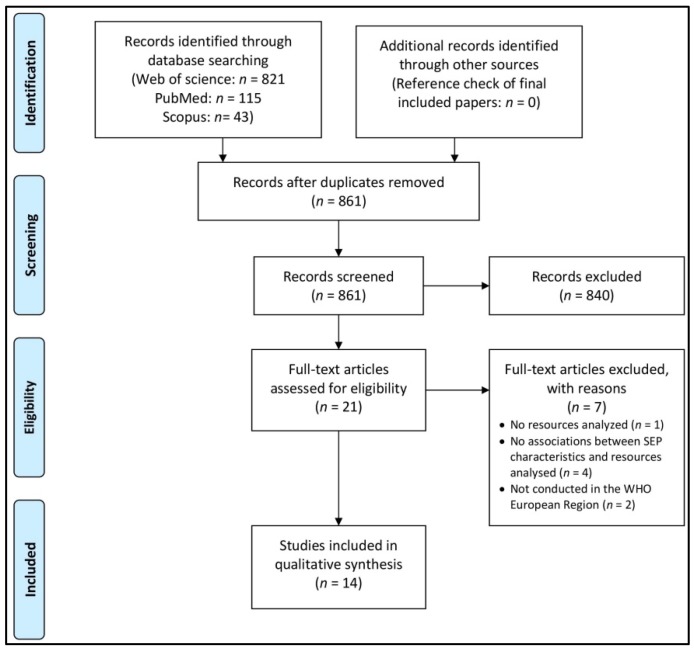
Flow diagram of study selection adapted from the PRISMA statements [28].

Two reviewers independently conducted the database search and removal of duplicates. All remaining titles and abstracts were screened by two reviewers independently to identify studies potentially meeting inclusion criteria. We calculated Cohen’s Kappa in order to assess inter-rater reliability agreement for title and abstract screening [33]. One reviewer retrieved and systematically assessed full texts of potentially eligible studies for inclusion, with a random sample check by a second reviewer. Any disagreements between the reviewers over the eligibility of particular studies were resolved by discussion and consultation of a third reviewer. Additionally, one reviewer screened the reference lists of all articles considered for data extraction in order to capture potential relevant publications missed by the electronic database searches. For illustrating the study selection process, we used the PRISMA guidelines [28] to produce a flow diagram (see Figure 1) and applied the PRISMA checklist (see Supplementary Materials Table S1).

One reviewer extracted data from all included studies using a predesigned and piloted data extraction form (see Supplementary Materials Table S2). A second reviewer performed cross-checks of data extraction. To compare studies concerning operationalisation of green and blue spaces, measurement of SEP and type of analysis, we summarised the following data (see Table 2): resources assessed with distance or availability measures; single SEP measures or indices applied; and environmental inequalities analysed in a descriptive, bivariate or multivariate manner. We defined distance measures as all access measures that incorporated objective proximity measures, such as distances to green or blue spaces from home address, and subjective proximity measures assessing perceived closeness to the next resources. Our definition of availability measures included objective and subjective measures, too. Objective availability incorporated proportions of resources in relation to a defined area size, such as buffers around the individual home address or census tracts. Subjective availability included measures describing perceived quantity of resources in the living environment. Furthermore, we grouped all studies according to their study design into ecological studies and cross-sectional studies on the individual level (see Table 2, Table 3 and Table 4).

In this review, we defined a descriptive analysis as a provision of quantitative resource measures across SEP groups in a cross table without performing a statistical test. We compared the highest to the lowest SEP group in order to assess if environmental inequalities exist or not. We did not define a specific cut-off concerning the magnitude of difference that had to be reached across social groups for being indicated as environmental inequalities because operationalisations of SEP and resources may be too heterogeneous. Bivariate analyses included all statistical methods analysing bivariate associations between socioeconomic characteristics and resources measures. Besides descriptive and bivariate results, we also extracted results from multivariate analyses. However, they may be difficult to compare because our research question does not consider a specific definition of additional adjustment or confounding factors. Multivariate models could therefore differ concerning their inclusion of other independent factors, and potential associations between SEP and resources may be concealed due to different modelling approaches. The following different multivariate approaches could be possible: (i) SEP indicators could be analysed as main independent variables with additional characteristics as confounders; (ii) SEP indicators could be only considered as confounders themselves in the context of other main variables of interest; and (iii) various SEP indicators could be considered for mutual adjustment in order to analyse the influence of every single indicator independently. As a result, multivariate models could be too heterogeneous to compare effect estimates between SEP dimensions and environmental resources. In both bivariate and multivariate analyses, results with a *p*-value < 0.05 were defined as statistically significant.

Our main goal was to capture findings from various studies reporting descriptive, bivariate or multivariate results in order to gain as much evidence as possible. Due to the heterogeneous study designs and analytical approaches, we were not able to apply a standardised quality assessment tool across studies. A further reason for not performing a quality assessment was that the main research question of many included studies was not on environmental inequalities; thus, results being relevant for our review were provided only as a complementary analysis.

We summarised associations between SEP and resources in two different ways. Firstly, we extracted whether environmental inequalities were detected within studies either in a descriptive, bivariate or multivariate analysis or if contrary associations were found within a study comparing several SEP dimensions. We further extracted the number of SEP indicators being analysed in each study (see Table 3).

Secondly, we summarised directions of associations grouped by SEP indicators. Per study, for each SEP indicator, one symbol per type of analysis (descriptive or bivariate/multivariate) was used to depict the association between the SEP indicator and environmental resources (see Table 4). We grouped bivariate and multivariate analyses and similar SEP indicators together in the table (e.g., measures of employment on the individual or aggregated level).

## 3. Results

The electronic database search identified 979 records. After duplicates were removed, 861 records were considered for title and abstract screening. Inter-rater reliability agreement of title and abstract screening was substantial (Cohen’s Kappa value of 0.77). We included 21 records into full-text analysis, from which seven were excluded. Fourteen studies met all inclusion criteria and were finally taken into account for qualitative synthesis (see Figure 1). No additional studies were identified by screening of the reference lists of the included studies.

### 3.1. Description of Studies

The majority of all studies (12 out of 14) analysed environmental inequalities related to green space [34,35,36,37,38,39,40,41,42,43,44,45] and two studies investigated unequal distributions of blue space [46,47]. Germany was the country where most studies were conducted (*n* = 8). Nine studies on green space analysed aggregated data with an ecological study design. All ecological studies analysed data on the small area scale, such as census tracts, neighbourhoods, or subdistricts (see Supplementary Materials Table S2). Four studies investigated cross-sectional data on the individual level and one study by Zandieh et al. performed both a cross-sectional analysis on the individual level and an ecological data analysis on the area level [40]. The majority of the studies calculated availability measures of green or blue space, especially those with an ecological study design, whereas cross-sectional studies also calculated distance-based measures. Furthermore, ecological analyses mainly applied socioeconomic deprivation indices, whereas studies on the individual scale analysed single SEP indicators.

Green space was mostly captured by combining various categories from existing land use categories of public green spaces, such as forests or parks. Some studies also calculated vegetation indices from remote sensing or other databases covering overall vegetation. One study applied a standardised audit tool to assess planting in parks [36]. One study on blue space also used existing land use data on urban blue [46], whereas the other study focused on aquatic environments localised by the study participants [47].

Bivariate statistics were most frequently applied for the analysis of relations between characteristics of SEP and environmental resources (*n* = 10 studies) and four studies applied multivariate methods (see Table 2). These four studies applied different modelling strategies, which makes them difficult to compare. One study mutually adjusted for seven SEP indicators and analysed their independent associations with two measures of green space with the additional consideration of city-levels as potential confounders [34]. On the contrary, another study analysed area deprivation as the dependent variable and considered six environmental measures of green space as independent variables [35]. The third study considered cities as potential effect modifiers on the pathway between SEP indicators (income and neighbourhood deprivation index) and two different operationalisations of green space [42]. The fourth study investigated relations between neighbourhood deprivation and various catchment areas of green space availability and adjusted for population density [45].

### 3.2. Associations between Indicators of SEP and Environmental Resources

Most of the ecological studies applied deprivation indices. Across ecological studies, there was a consistent trend that deprived areas had less resources available than more affluent areas. In cross-sectional studies on the individual level, where mostly various single SEP indicators were considered, heterogeneous results on social inequalities in resources were found. These heterogeneous results were independent of the applied analysis method (descriptive, bivariate or multivariate analyses) (see Table 3 and Table 4). Measures of low education showed, for example, consistent relations with a smaller amount of resources or greater distances to resources, whereas age relations were found in terms of old people having more green space available or shorter distances to green spaces than young people.

Contrasting associations were found either for the same SEP indicator across different environmental measures (distance versus availability measure) or across different SEP indicators for the same environmental measure (see Supplementary Materials Table S2). For example, the study by Wüstemann et al. on urban blue reported, in both descriptive and bivariate analyses, that people with a migration background had a lower amount of urban blue space available in a 500 m radius around the household. However, when the distance to blue spaces was additionally considered, this study also found a significant association between migration background and a lower distance to blue spaces. This study showed contrasting associations for the same social dimension across two different environmental outcome measures: one that measured availability and one that calculated distances [46]. The study by Laatikainen et al. is an example where different SEP indicators showed contrary associations for the same environmental measure. People with low income, no car, and no home ownership had higher distances to blue space, whereas old people and people with no employment had shorter distances [47].

## 4. Discussion

This systematic review summarised study results on the social distribution of environmental resources across the WHO European Region since 2010. Ecological studies showed a consistent trend that socioeconomically deprived areas have fewer resources available than more affluent areas. Associations in cross-sectional studies on the individual level were mixed and dependent on the type of SEP indicator and applied environmental measures.

Different results between ecological and cross-sectional studies on the individual level could be explained by the different SEP measures applied in both kinds of study designs. Ecological studies mostly analysed socioeconomic deprivation indices combining various SEP indicators, whereas cross-sectional studies focused on single dimensions of SEP.

In cross-sectional studies, measures of low education and low income were often associated with measures of environmental resources, indicating lower access or availability. Education and income are regarded as sound determinants for material resources and could therefore adequately explain social inequalities in environmental resources, as material circumstances could determine where people live [48].

Despite the fact that deprivation indices of included studies incorporated different numbers and types of socioeconomic factors, results on environmental inequalities were consistent across studies. Deprivation indices might therefore be appropriate measures to analyse general trends of environmental inequalities related to resources within and between cities on the small area scale. However, there is evidence from only one study whether a deprivation index showed environmental inequalities when applying it on the individual level [42]. Cross-sectional studies mostly applied single SEP indicators and did not consider aggregated SEP measures on the small area scale. Environmental inequality studies on the aggregated scale are further prone to the Modifiable Area Unit Problem (MAUP) [49]. There is evidence that when the geographic unit of the analysis is changed, such as from census tract to blocks, contrasting results may occur.

There are further issues when evidence of included studies was compared. Firstly, there is still no common operationalisation of green and blue spaces. Mostly, land use categories from national or local land use plans are combined to characterise green areas, such as public parks or forests [34,38,40,50]. Other studies used data from remote sensing to capture urban vegetation, such as with the Normalized Difference Vegetation Index [42,43]. For the two studies on blue space, different operationalisation measures were used. One study used land use categories from the European Atlas [46] and the other focused on overall aquatic environments that were used and localised by the study participants [47].

Secondly, one further central result of our review was that relations between SEP indicators and environmental resources were often dependent on the operationalisation of resource measures, e.g., if availability or distance measures were analysed [34,46]. This is in accordance with a systematic review by Rigolon focusing on environmental inequalities of urban parks in mainly industrialised countries. One central result of this review for measures of ethnicity was that ethnic minority groups have shorter distances to parks, whereas for other SEP indicators associations were mixed and both positive and negative associations were found. For quantity measures, results on inequalities were more consistent, meaning that minority groups and low SEP groups have a lower quantity of parks than other groups. [25]. In our review, mixed associations were also predominantly found in cross-sectional studies where both distance and availability measures of green and blue spaces were analysed in relation to single SEP indicators [34,46].

When availability of resources was measured on the individual level, there was a great heterogeneity of buffers, which were used to define the catchment area of green or blue spaces. Buffers of 400 m, 500 m, 800 m, 1000 m and 2000 m were defined across studies, which made comparisons of study results challenging. In ecological studies, green space availability was mostly assessed based on administrative boundaries. There is still no theoretical assumption on the distance that should be applied to assess green space availability both on the individual and aggregated scale in order to analyse environmental inequalities or relations to health and health behaviours. Most health-related studies used a maximum walking distance of 800 m around individual home addresses for reaching resources or other facilities [51,52,53,54]. A systematic review by Browning and Lee examined how various catchment areas of green spaces influenced associations to health outcomes. One central result was that predictive power for associations between green space measures and health decreased when studies applied buffers greater than 2000 m, which indicates a dosage effect of green spaces and their influence towards individual health [55].

Thirdly, almost all identified studies in the WHO European Region assessed resource availability with objective measures. However, it is important to mention that objective measures do not necessarily reflect subjective measures. Studies showed that perceived green space availability is more strongly associated with walking or use of green than objective measures [56,57]. A study conducted in Dortmund, Germany, further indicated that individuals with a low SEP were more likely to feel subjectively annoyed by the same magnitude of objective environmental measures than individuals with a high SEP. Relations for noise indicated that individuals with a low SEP had a higher chance to feel subjectively annoyed by objectively measured noise levels ≥55 dB than individuals with a high SEP. For green space, no consistent trend of a modifying role of SEP was found, however, presumably due to the low sample size in this study [58]. Nevertheless, such results indicate that environmental inequalities assessed with objective measures could potentially underestimate the health impact of inequalities among people with a low SEP as people with a low SEP could be more sensitive to the same objective exposure level than people with a high SEP.

Most of these issues mentioned above are also found when looking at studies from industrialised countries outside the WHO European Region. They indicate that various operationalisations of green spaces, including distance versus availability measures, various types of green, but also quality aspects, are important to consider when environmental inequalities are assessed in order to prevent false conclusions.

Evidence from countries outside the WHO European Region suggests that results on environmental inequalities of resources based on distance-based measures should be treated with caution as further important quality aspects could be neglected. Studies from the United States and Australia found that areas with a low income or a high amount of minorities have shorter distances to public green spaces [59,60,61]. However, when taking other aspects of green spaces into account, these studies identified that, apart from shorter distances, these public green spaces have a lower quality [59] or were smaller [59,61]. Another study reported that, in contrast to shorter distances to green spaces, overall green space coverage in the neighbourhood was smaller for deprived areas [60]. 

Furthermore, in comparison to ecological studies identified in the WHO European Region, ecological studies from Australia, Canada, or the United States applied deprivation indices less often and considered single SEP measures, such as income or ethnicity, instead [62,63,64]. Across these studies, measures of low income were consistently associated with lower availability of green space, which was also mostly the case in cross-sectional studies on the individual level in the WHO European Region identified by this review.

Concerning operationalisation and definition of green spaces, it could be further important to distinguish between private and public green spaces when environmental inequalities are analysed. For the city of Sydney, Lin et al. found that areas with a low SEP have more public green space than high SEP areas; however, they have less private green space [65]. Therefore, high SEP areas could still have more overall green space when considering both green space categories.

This review provides good starting points for further research in this field, especially concerning operationalisations of resources, choice and types of SEP indicators, and appropriate study designs for analysing environmental inequalities. Firstly, most studies included in this review were conducted in Germany. Therefore, it is not possible to make general statements on the distribution of resources between social groups in the whole WHO European Region and more comparable studies are needed within and across European countries.

Secondly, there is a further need for longitudinal studies that analyse to what extent potential environmental green space inequalities change over time. One of the first longitudinal studies was published in 2017 by Casey et al., who analysed how inequalities of green space have changed over 10 years in the United States. A central result of this study was that areas with a higher amount of minorities lost greenness over the years, whereas greenness increased in areas with higher percentages of whites [64].

Thirdly, future studies on relations between resources and health or between SEP and resource access should develop comparable distance measures or catchment areas that adequately represent the resource access of the target population. It is important that future studies consider different components of resource measures, such as distance, availability or quality, because they have also different implications for public health [25].

Finally, there is a need for further research in the WHO European Region on the extent to which the strength of associations between SEP and environmental resources are influenced by the type of measure applied: subjective or objective.

### 4.1. Limitations

Our review visualised the directions of associations between SEP indicators and green or blue space measures and if they were significant or not. The different applied analytical methods (description versus correlation versus regression) and the different operationalisations of SEP characteristics (continuous variables versus categorical variables with different numbers of categories) across studies prevented any comparisons of the magnitude of the inequalities.

Moreover, we referred to the results of statistical tests as reported in the studies, mostly dichotomising the calculated *p*-values with the threshold 0.05 to assess statistical significance. However, this approach has been criticised [66,67]. *P*-values alone are not sufficient to assess the scientific relevance of study results and therefore should be interpreted with caution.

Furthermore, analysing potential mechanisms leading to environmental inequalities was not the main target of this review. Only a few studies discussed potential mechanisms, such as aspects of procedural justice [38] or specific urban/rural characteristics [42]. A comprehensive assessment of mechanisms leading to social inequalities in environmental resources would need another kind of systematic review.

As a final limitation, we did not perform a separate search in sources of grey literature. This approach was supported by the fact that we did not identify any relevant grey literature source when all references of included studies were checked.

### 4.2. Strengths

One of the main strengths of this review is that there was no restriction on specific study designs or environmental measures in order to assess evidence on environmental inequalities of green or blue space in its broadest way. Furthermore, this review systematically considered a wide range of sociodemographic and socioeconomic dimensions according to the PROGRESS-Plus framework. To the best of our knowledge, this is the first review to systematically assess evidence on this topic in the WHO European Region since the year 2000.

### 4.3. Implications for Practice

Our review showed that aspects of equality should be an integrative part in the context of green space planning and green space interventions. In a recent WHO review on urban green space interventions, strategies are provided on how aspects of equity could be considered best in the process of healthy green space planning for addressing avoidable social differences in green space availability and accessibility. In order to target interventions in the most equitable way, two main recommendations are the development of adequate equity indicators in collaboration with local planners and other relevant actors, and the engagement of vulnerable or disadvantaged groups in a participation process [68]. For green space interventions, it is further important to take into account the risk of gentrification processes because green revitalisation of an area might also result, in the long-term, in only people with a high SEP being able to afford to live there [69]. A promising approach in the context of environmental gentrification is the strategy ‘just green enough’, which aims to replace market-driven processes with bottom-up processes that address green space interventions that involve the needs and concerns of the local community [23,70].

## 5. Conclusions

Despite data limitations, this review has shown that social inequalities in green space access and availability are an issue in the WHO European Region. On the area level, environmental inequalities in resources were consistently found, and results on the individual level were dependent on the type of SEP indicator and the applied environmental resource measure. In order to generate stronger systematic evidence in this field, more Europe-wide studies based on comparable methods are needed. An integrative monitoring of environmental inequalities would guarantee a more comprehensive and comparable data basis and would offer the possibility to analyse trends over time.

## Figures and Tables

**Table 1:** Search terms and Medical Subject Headings in PubMed.

Search	Query
#1	(“sociological factors”[MeSH Terms] OR disadvantaged[All Fields] OR disadvantage[All Fields] OR deprived[All Fields] OR social[All Fields] OR socio*[All Fields] OR "vulnerable populations"[MeSH Terms] OR vulnerable[All Fields] OR vulnerability[ALL Fields] OR psychosocial[All Fields] OR psycho-social[All Fields] OR “socioeconomic factors”[MeSH Terms] OR socio-economic[ALL Fields] OR deprivation[All Fields] OR socio-demographic[All Fields])
#2	(“green space” [Title/Abstract] OR “green spaces” [Title/Abstract] “open space” [Title/Abstract] OR “open spaces” [Title/Abstract] OR “natural space”[Title/Abstract] OR “natural spaces”[Title/Abstract] OR “green environment” [Title/Abstract] OR “green environments” [Title/Abstract] OR “green area” [Title/Abstract] OR “green areas” [Title/Abstract] OR greenery [Title/Abstract] OR greenness [Title/Abstract] OR “urban green” [Title/Abstract] OR “public green” [Title/Abstract] OR “neighbourhood green” [Title/Abstract] OR “neighborhood green” [Title/Abstract] OR “natural environment” [Title/Abstract] OR “natural environments” [Title/Abstract] OR park [Title/Abstract] OR parks [Title/Abstract] OR forest [Title/Abstract] OR forests [Title/Abstract] OR “urban park” [Title/Abstract] OR “urban parks” [Title/Abstract] OR “city park” [Title/Abstract] OR “city parks” [Title/Abstract] OR “park access” [Title/Abstract] OR “public garden” [Title/Abstract] OR “public gardens” [Title/Abstract] OR “blue space”[Title/Abstract] OR “blue spaces”[Title/Abstract] OR “blue area” [Title/Abstract] OR “blue areas” [Title/Abstract]OR beach[Title/Abstract] OR beaches[Title/Abstract] OR lake [Title/Abstract] OR lakes [Title/Abstract]OR river [Title/Abstract] OR rivers [Title/Abstract] OR sea [Title/Abstract] OR “recreational space” [Title/Abstract] OR “recreational spaces” [Title/Abstract] OR “recreational area” [Title/Abstract] OR “recreational areas” [Title/Abstract] OR outdoor [Title/Abstract])
#3	(inequality[Title/Abstract] OR inequity[Title/Abstract] OR inequities[Title/Abstract] OR inequalities[Title/Abstract] OR unequal[Title/Abstract] OR "environmental justice"[Title/Abstract] OR "environmental injustice"[Title/Abstract])
#4	(“2010/01/01”[Date—Publication]: “2017/12/31”[Date—Publication])
Final search	#1 AND #2 AND #3 AND #4
Additional filters	Language: EnglishSpecies: Humans

**Table 2:** Characteristics of studies.

Author, Year	Green and Blue Space Operationalisation		SEP Indicator		Type of Environmental Inequality Analysis			Country
	Distance Measure	Availability Measure	Single	Index	Descriptive	Bivariate	Multivariate	
**Cross-sectional studies blue space**								
Wüstemann, 2017 [46]	x	x	x		x	x		Germany
Laatikainen, 2015 [47]	x		x			x		Finland
**Cross-sectional studies green space**								
Wüstemann, 2017 [34]	x	x	x		x		x	Germany
Zandieh, 2017 [40]		x	x			x		U.K.
Markevych, 2017 [42]		x	x	x			x	Germany
**Ecological studies green space**								
Hoffimann, 2017 [35]	x	x		x	x	x	x	Portugal
Kabisch, 2014 [38]		x	x		x			Germany
Kabisch, 2016 [39]		x	x	x		x		Germany
Zandieh, 2017 [40]		x		x		x		U.K.
Padilla, 2016 [41]		x		x		x		France
Lakes, 2014 [43]		x		x		x		Germany
Flacke, 2016 [44]		x	x			x		Germany
Schüle, 2017 [45]		x		x			x	Germany
Gallo, 2015 [36]		x		x		x		U.K.
Cohen, 2012 [37]		x	x			x		France

**Table 3:** Summary of relationships between socioeconomic position (SEP) and environmental resources across studies, and number of analysed SEP indicators.

Studies Grouped by Analysis Type	Descriptive	Bivariate	Multivariate	Number of SEP Indicators (*n*)
**Cross-sectional analyses blue space**				
Wüstemann, 2017 [46]	↕	↕		6
Laatikainen, 2015 [47]		↕		6
**Cross-sectional analyses green space**				
Wüstemann, 2017 [34]	↕		↕	7
Zandieh, 2017 [40]		↓		1
Markevych, 2017 [42]			↕	2 *
**Ecological analyses green space**				
Hoffimann, 2017 [35]	↓	↓	↓	1 *
Kabisch, 2014 [38]	↕			2
Kabisch, 2016 [39]		↓		3 *
Zandieh, 2017 [40]		↓		1 *
Padilla, 2016 [41]		↓		1 *
Lakes, 2014 [43]		↓		1 *
Flacke, 2016 [44]		↓		1
Schüle, 2017 [45]			↓	1 *
Gallo, 2015 [36]		-		1 *
Cohen, 2012 [37]		-		1

↓ Low SEP groups have lesser amount of resources available or greater distances to resources compared to high SEP groups (This association was detected at least for one SEP indicator); ↕ SEP indicator(s) showed contrary directions of associations within study either for the same SEP indicator across different environmental resource measures or across different SEP indicators for the same environmental resource measure; − no social inequalities found; * at least one deprivation index analysed

**Table 4:** Summary of relationships between measures of SEP and environmental resources.

SEP Dimension	Individual Data Analyses	Ecological Data Analyses
High deprivation (Index) [35,36,39,40,41,42,43,45]	**↕** *	↓ * **↓** * ↓ ↓ ↓↓* **- -**
Low income [34,37,42,46,47]	↑ ↓ **↓** * **↕** * **↕** * **-**	**-**
Low education [34,46]	↓ ↓ **↓** * **-**	
No employment [34,44,46,47]	**↓** ↑* ↑* **- -**	**↓**
With migration background [34,38,46]	↓* ↕ **↕ -**	↓
Foreign nationality [34,39,46]	↕ ↕ **↕ -**	**↓** *
Black and minority ethnic groups [40]	**↓**	
Gender: female [34,47]	↓ * **↑** *	
Old age [34,38,47]	↑ ↑* **↑** *	↑
With children in household [34]	↓ * **↓** *	
Single parent household [39]		**-**
No car [47]	↕*	
No home ownership [47]	**↕** *	

↓ Descriptive Analysis: low SEP groups have a lesser amount of resources available or greater distances to resources compared to high SEP groups; **↓** Bivariate/Multivariate Analysis: low SEP groups have a lesser amount of resources available or greater distances to resources compared to high SEP groups; ↑ Descriptive Analysis: low SEP groups have a greater amount of resources available or shorter distances to resources compared to high SEP groups; **↑** Bivariate/Multivariate Analysis: low SEP groups have a greater amount of resources available or shorter distances to resources compared to high SEP groups; **-** Bivariate/Multivariate Analysis: no social inequalities found; ↕ Descriptive Analysis: SEP indicator showed contrary directions within study (different operationalisations for resources were analysed within studies); **↕** Bivariate/Multivariate Analysis: SEP indicator showed contrary directions within study (different operationalisations for resources were analysed within studies); * Association not found in each subanalysis within study where different operationalisations for resources were applied

## Supplementary Materials

The following are available online at https://www.mdpi.com/1660-4601/16/7/1216/s1, Table S1: PRISMA checklist, Table S2: Data extraction, S3: Search terms from Web of Science and Scopus.
