# Supplementary material for: Social Inequalities in Environmental Resources of Green and Blue Spaces: A Review of Evidence in the WHO European Region
Source: Int J Environ Res Public Health. 2019 Apr 4;16(7):1216. doi: 10.3390/ijerph16071216 (PMC6480666; doi:10.3390/ijerph16071216)
Supplement: Supplementary file 1 [file ijerph-16-01216-s001.pdf]

Supplement S1: PRISMA checklist

Section/topic	#	Checklist item	Reported on page #
**TITLE**			
Title	1	Identify the report as a systematic review, meta-analysis, or both.	1
**ABSTRACT**			
Structured summary	2	Provide a structured summary including, as applicable: background; objectives; data sources; study eligibility criteria, participants, and interventions; study appraisal and synthesis methods; results; limitations; conclusions and implications of key findings; systematic review registration number.	1
**INTRODUCTION**			
Rationale	3	Describe the rationale for the review in the context of what is already known.	1,2
Objectives	4	Provide an explicit statement of questions being addressed with reference to participants, interventions, comparisons, outcomes, and study design (PICOS).	2
**METHODS**			
Protocol and registration	5	Indicate if a review protocol exists, if and where it can be accessed (e.g., Web address), and, if available, provide registration information including registration number.	2
Eligibility criteria	6	Specify study characteristics (e.g., PICOS, length of follow-up) and report characteristics (e.g., years considered, language, publication status) used as criteria for eligibility, giving rationale.	2,3,4
Information sources	7	Describe all information sources (e.g., databases with dates of coverage, contact with study authors to identify additional studies) in the search and date last searched.	2,3,4
Search	8	Present full electronic search strategy for at least one database, including any limits used, such that it could be repeated.	3
Study selection	9	State the process for selecting studies (i.e., screening, eligibility, included in systematic review, and, if applicable, included in the meta-analysis).	3,4
Data collection process	10	Describe method of data extraction from reports (e.g., piloted forms, independently, in duplicate) and any processes for obtaining and confirming data from investigators.	4,5
Data items	11	List and define all variables for which data were sought (e.g., PICOS, funding sources) and any assumptions and simplifications made.	2,3,4,5
Risk of bias in individual studies	12	Describe methods used for assessing risk of bias of individual studies (including specification of whether this was done at the study or outcome level), and how this information is to be used in any data synthesis.	5
Summary measures	13	State the principal summary measures (e.g., risk ratio, difference in means).	n.a.
Synthesis of results	14	Describe the methods of handling data and combining results of studies, if done, including measures of consistency (e.g., I^2) for each meta-analysis.	n.a.

Section/topic	#	Checklist item	Reported on page #
Risk of bias across studies	15	Specify any assessment of risk of bias that may affect the cumulative evidence (e.g., publication bias, selective reporting within studies).	n.a.
Additional analyses	16	Describe methods of additional analyses (e.g., sensitivity or subgroup analyses, meta-regression), if done, indicating which were pre-specified.	n.a.
**RESULTS**			
Study selection	17	Give numbers of studies screened, assessed for eligibility, and included in the review, with reasons for exclusions at each stage, ideally with a flow diagram.	5
Study characteristics	18	For each study, present characteristics for which data were extracted (e.g., study size, PICOS, follow-up period) and provide the citations.	5,6,7,8 Supplements
Risk of bias within studies	19	Present data on risk of bias of each study and, if available, any outcome level assessment (see item 12).	n.a.
Results of individual studies	20	For all outcomes considered (benefits or harms), present, for each study: (a) simple summary data for each intervention group (b) effect estimates and confidence intervals, ideally with a forest plot.	n.a.
Synthesis of results	21	Present results of each meta-analysis done, including confidence intervals and measures of consistency.	n.a.
Risk of bias across studies	22	Present results of any assessment of risk of bias across studies (see Item 15).	n.a.
Additional analysis	23	Give results of additional analyses, if done (e.g., sensitivity or subgroup analyses, meta-regression [see Item 16]).	n.a.
**DISCUSSION**			
Summary of evidence	24	Summarize the main findings including the strength of evidence for each main outcome; consider their relevance to key groups (e.g., healthcare providers, users, and policy makers).	8,9,10
Limitations	25	Discuss limitations at study and outcome level (e.g., risk of bias), and at review-level (e.g., incomplete retrieval of identified research, reporting bias).	10,11
Conclusions	26	Provide a general interpretation of the results in the context of other evidence, and implications for future research.	11
**FUNDING**			
Funding	27	Describe sources of funding for the systematic review and other support (e.g., supply of data); role of funders for the systematic review.	11

n.a. = not applicable

From: Moher D, Liberati A, Tetzlaff J, Altman DG, The PRISMA Group (2009). Preferred Reporting Items for Systematic Reviews and Meta-Analyses: The PRISMA Statement. PLoS Med 6(7): e1000097. doi:10.1371/journal.pmed1000097

For more information, visit: **www.prisma-statement.org**.

Supplement S2: Description of studies and reported associations on social inequalities in environmental resources

Author, year	*Place of study*	*Unit of analysis, study population and sample size*	**Study type**	*Operationalization of green or blue space*	*Socioeconomic and sociodemographic characteristics*	*Type of analysis*	*Results on environmental inequalities in symbols*
Cross-sectional studies blue space							
Wüstemann, 2017 (Journal: Ecological Indicators)	Germany, 53 major cities	**Individual level:** Adults (N=4588) from the German Socio-Economic Panel	Cross-sectional	**Objective:** Urban blue (visible water bodies and courses >1 ha from European Urban Atlas)<br>**Operationalization:** Euclidian distance to blue space from household; Amount of urban blue in a 500 m buffer around the household.	Income, education, employment, migration background, German nationality, child in household	**Description:** Crosstables<br>**Bivariate analysis**: T-test and F-test to compare mean values of urban blue across socioeconomic groups	**Description:**<br>*Amount of urban blue:*<br>⊖ (with migration background; with children in household; no German nationality; low education (n.l. in middle groups))<br>⊕ (no employment, low income (n.l. in middle groups))<br>*Distance to urban blue:*<br>⊖ (low education (n.l. in middle groups)<br>⊕ (with migration background, low income (n.l. in middle groups), no German nationality<br>= (employment, child in household)<br>**Bivariate:**<br>*Amount of urban blue :*<br>⊖ (with migration background, no German nationality, with children in household)<br>n.s. (employment, income, education)<br>*Distance to urban blue:*<br>⊕ (with migration background, no German nationality)<br>n.s. (employment, income, education, with children in household)
Laatikainen, 2015	Finland, Helsinki Metropolitan Area	**Individual level:** Adults between 15 and 75 years old (N=2031)	Cross-sectional)	**Subjective:** Location of used aquatic environments were marked by study participants.<br>**Operationalisation (objective):** Euclidean distance to nearest water, Euclidean distance to aquatic activity point, travel distance to activity point, travel time to activity point	Age, gender, employment status, income, car ownership, home ownership	**Bivariate analysis**: Mann-Whitney U test	**Bivariate:**<br>*Distance to nearest water*<br>⊖ (low income, no car, no home ownership)<br>⊕ (no employment status, age (≥65 years)[1)])<br>n.s. (gender)<br>*Distance to aquatic activity point*<br>⊕ (sex (female)[2)], no employment status, no car, no home ownership)<br>n.s. (income, age (≥65 years))<br>*Travel distance to activity point*<br>⊕ (no employment status, low income, no car, no home ownership)<br>n.s. (age (≥65 years), sex)<br>*Travel time to activity point*<br>⊖ (low income)<br>n.s. (employment status, age (≥65 years), sex, car ownership, home ownership)

Cross-sectional studies green space							
Wüstemann, 2017 (Journal: Landscape and Urban Planning)	Germany, 53 major cities	**Individual level:** Adults (N=4588) from the German Socio-Economic Panel	Cross-sectional	**Objective:** Green urban areas and forests (land use categories from European Urban Atlas)<br>**Operationalisation:** Euclidian distance to green from household; Amount of urban green in a 500 m buffer around household	Income, age, gender, education, employment, migration background, German nationality	**Description:** Crosstables<br>**Multivariate analysis**: multiple linear regression adjusted for city	**Description:**<br>*Amount of green:*<br>⊖ (with migration background, low income (n.l. in middle groups), low education, no employment, no German nationality)<br>⊕ (age (≥65 years))<br>= (gender)<br>*Distance to green:*<br>⊖ (low income (n.l. in middle groups), no employment, low education (n.l. in middle groups), gender (female)<br>⊕ (age (≥65 years) (n.l. in middle groups), no German nationality)<br>= (migration background)<br>**Multivariate:**<br>*Amount of green space:*<br>⊖ (low income, low education)<br>⊕ (age (≥65 years), children in household)<br>n.s. (migration background, German nationality, no employment)<br>*Distance to green:*<br>n.s. (for all socioeconomic measures)
Zandieh, 2017	UK (city of Birmingham)	**Individual level:** Adults ≥65 years (n=173)	Cross-sectional study	**Objective:** Green space (land use data comprising public parks and gardens, natural green spaces, amenity green spaces)<br>**Operationalisation:** Percentage of green space in a 2 km buffer around the home address**.**	Ethnicity	**Bivariate:** Pearson´s correlation coefficient	**Bivariate:**<br>*Percentage of green space:*<br>⊖ (black and minority ethnic groups)
Markevych, 2017	Germany (city of Munich Leipzig, Bad Honnef and Wesel)	**Individual level:** Parents (Munich (n=1865); Leipzig (n=337); Bad Honnef (n=155); Wesel (n=1439))	Cross-sectional study	**Objective:** Green space based on **r**emote sensing data (Normalized differenced vegetation index (NDVI) and tree cover)<br>**Operationalisation:** Mean NDVI and percent of tree cover in a 500 and 1000 m buffer around home address	Household income (individual level); German Deprivation index on municipality level	**Multivariate:** linear regression analysis by city. Cities are considered as effect modifiers on the pathway between SEP and green space. Simultaneous consideration of income and deprivation index adjusted further for number of children and study type	**Multivariate:**<br>*NDVI (both buffers)*<br>⊖ (low income (Munich, Leipzig); high deprivation (Munich, Wesel))<br>⊕ (low income (Wesel))<br>n.s. (income (Bad Honnef); high deprivation (Bad Honnef, Leipzig))<br>*Tree cover (both buffers)*<br>⊖ (low income (Munich); high deprivation (Munich)<br>⊕ (high deprivation (Wesel))<br>n.s. (income (Leipzig, Bad Honnef, Wesel); high deprivation (Bad Honnef, Leipzig))

Ecological studies green space							
Hoffimann, 2017	Portugal (city of Porto)	**Aggregated level:** census tracts (N=2064)	Ecological study	**Objective:** Public green spaces (N=55) from the Porto city council<br>**Operationalisation:** Availability of green space (Yes/No) within 800 m road distance from neighbourhood centroid; Mean distance to green spaces within 800 m; Number of green spaces within 800 m; Amount of green spaces per inhabitant within 800 m.	Deprivation index	**Description:** Crosstables<br>**Bivariate:** ordinal regression<br>**Multivariate:** Ordinal regression (Dependent variable: Deprivation Index; Independent variables: Green space variables and quality indicators of green spaces, (environmental quality, amenities, safety)	**Description:**<br>*Availability of green space (yes vs. no) within 800 m road distance*<br>⊖ (high deprivation)<br>*Number of green spaces:*<br>⊖ (high deprivation (n.l. in middle groups))<br>*Distance to green spaces:*<br>⊖ (high deprivation(n.l. in middle groups))<br>*Amount of green spaces per inhabitant:*<br>⊖ (high deprivation(n.l. in middle groups))<br>**Bivariate:**<br>*Availability of green space*<br>⊖ (high deprivation)<br>*Number of green spaces:*<br>⊖ (high deprivation)<br>*Distance to green spaces:*<br>⊖ (high deprivation)<br>*Amount of green spaces per inhabitant:*<br>n.s. (Deprivation Index)<br>**Multivariate:**<br>*Availability of green space*<br>n.s. (deprivation index)<br>*Number of green spaces:*<br>n.s. (deprivation index)<br>*Distance to green spaces:*<br>⊖ (high deprivation)<br>*Amount of green spaces per inhabitant:*<br>n.s. (deprivation index)
Kabisch, 2014	Germany (city of Berlin)	**Aggregated level:** sub-districts (n=60); three spatial clusters from cluster analysis (n=28; n=9; n=23)	Ecological study	**Objective:** Green space per sub-district (land use data comprising forests, parks, cemeteries, allotment gardens, brownfields with vegetation)<br>**Operationalisation:** Percentage per sub-district	Percentage of immigrants (three spatial cluster categories); percentage of individuals ≥65 years (three spatial cluster categories)	**Description:** Crosstables (prevalence of urban green and the two socioeconomic factors across the three clusters) ; Figures (Lorenz curve based on calculation of the GINI coefficient	**Description crosstable:**<br>*Percentage of green space:*<br>⊖ (high amount of immigrants (n.l. in middle groups))<br>⊕ (age (high amount of inhabitants ≥65 years))<br>**Description Lorenz curve:**<br>*Percentage of green space:*<br>⊖ (high amount of immigrants)<br>⊕ (age (high amount of inhabitants ≥65 years))
Kabisch, 2016	Germany (city of Berlin)	**Aggregated level:** sub-districts (n=60)	Ecological study	**Objective:** Natural areas (land use data comprising forests, urban green and parks, cemeteries, allotment gardens, waterbodies (lakes, rivers, canals)	Social status index of parents; percentage of children living in single parent households;	**Bivariate:** Spearman´s correlation coefficient	**Bivariate:**<br>*Percentage of natural areas*<br>n.s. (social status index, non-German, single parent households)<br>*m^2 of natural areas per inhabitant*<br>⊖ (non-German)<br>n.s. (social status index, single parent households)

				**Operationalisation:** Percentage of natural areas, m^2 of natural areas per inhabitant, availability (percentage of inhabitants living a maximum of 300 m distance away from a natural area)	Percentage of children with background other than German		*Availability of natural areas*<br>⊖ (low social status)<br>n.s. (non-German, single parent households)
Zandieh, 2017		**Aggregated level:** Combination of electoral wards (n=2; Low deprivation area vs. high deprivation area)	Ecological study	**Objective:** Green space (land use data comprising public parks and gardens, natural green spaces, amenity green spaces)<br>**Operationalisation:** Percentage of green space per area	Deprivation Index	**Bivariate:** t-test	**Bivariate:**<br>*Percentage of green space:*<br>⊖ (high deprivation)
Padilla, 2016	France (Nice metropolitan area)	**Aggregated level:** census tracts (N=236)	Ecological study	**Objective:** Green space (land use data comprising natural areas)<br>**Operationalisation:** Percentage of green space per census tract	Deprivation Index	**Bivariate:** Spearman´s correlation coefficient	**Bivariate:**<br>*Percentage of green space:*<br>⊖ (high deprivation)
Lakes, 2014	Germany (city of Berlin)	**Aggregated level:** Planning units (N=434)	Ecological study	**Objective:** Green space based on remote sensing data (Normalized differenced vegetation (NDVI) index<br>**Operationalization:** Aggregated mean NDVI per planning unit	Deprivation Index	**Bivariate:** Pearson´s correlation coefficient	**Bivariate:**<br>*Mean NDVI:*<br>⊖ (high deprivation)
Flacke, 2016	Germany (city of Dortmund)	**Aggregated level:** neighbourhoods (n=170)	Ecological study	**Objective:** green space (land use data comprising parks and forests >1 ha)<br>**Operationalisation:** Percentage of green space including green spaces in a 400 m buffer around the neighbourhood	Percentage of people of the total neighbourhood population receiving unemployment benefits or social welfare	**Bivariate:** Spearman´s correlation coefficient	**Bivariate:**<br>*Percentage of green space:*<br>⊖ (higher amount of people receiving unemployment benefits or social welfare)
Schüle, 2017	Germany (city of Munich)	**Aggregated level:** neighbourhoods (n=108)	Ecological study	**Objective:** green space (land use data comprising public parks and forests)<br>**Operationalisation:** Percentage of green space within and around neighbourhoods (five buffers	Deprivation index	**Multivariate:** Log-gamma regression from the group of generalized linear models adjusted for population density	**Multivariate:**<br>*Percentage of green space within and around neighbourhoods*<br>⊖ (high deprivation (200 m - 1000 m buffer)<br>n.s. (deprivation index (no buffer)<br>*Percentage of green space around neighbourhood centroids*<br>⊖ (high deprivation (for all radii))

				from 200 m up to 1000 m): Percentage of green space around neighbourhood centroids (1000 m, 1500 m, 2000 m, 2500 m, and 3000 m radii)			
Gallo, 2015	UK (city of Newcastle)	**Aggregated level:** comparison of two parks in a deprived and in an affluent area	Ecological study	**Objective:** Observational Park Audit Tool<br>**Operationalisation:** Presence and density of planting	Two socioeconomic disparate areas were selected based on Indices of Multiple Deprivation	**Bivariate:** Chi2 test to compare distributions between the two parks	**Bivariate:**<br>*Presence and density of planting*<br>n.s. (deprived vs. affluent park)
Cohen, 2012	France (city of Paris)	**Aggregated level:** census blocks (n=282 with botanical information)	Ecological study	**Objective:** FLORA database (n=282 polygons containing more than three botanical species and vegetal species which more than two occurrences	Mean household income	**Description:** descripted in text (no tables, figures , etc. provided)<br>**Bivariate:** Linear Correlation analysis (scatter plot)	**Bivariate:**<br>*Number of species:*<br>n.s. (household income)

„=“ = no social unequal distribution of green space
n.s. = not significant
„⊖“ = Hypothesis supported: low SEP groups have lower resources available or greater distances to resources compared to high SEP groups / significant association in correlation or multivariate analysis (p-value < 0.05)
„⊕“ = Hypothesis challenged/not supported: low SEP groups have more resources available or lower distances to resources compared to high SEP groups / significant association in correlation or multivariate analysis (p-value < 0.05)
n.l. = non-linear
[1)] age: old people versus people of young/middle age as reference
[2)] sex: females versus males as reference

Supplement S3: Search terms for Web of Science and Scopus

Web of Science

TS=(disadvantaged OR disadvantage OR deprived OR social OR socio* OR vulnerable OR vulnerability OR psychosocial OR psycho-social OR socio-economic OR deprivation OR socio-demographic)

AND

TS=(“green space” OR “green spaces” “open space” OR “open spaces” OR “natural space” OR “natural spaces” OR “green environment” OR “green environments” OR “green area” OR “green areas” OR greenery OR greenness OR “urban green” OR “public green” OR “neighbourhood green” OR “neighborhood green” OR “natural environment” OR “natural environments” OR park OR parks OR forest OR forests OR “urban park” OR “urban parks” OR “city park” OR “city parks” OR “park access” OR “public garden” OR “public gardens” OR “blue space” OR “blue spaces” OR “blue area” OR “blue areas” OR beach OR beaches OR lake OR lakes OR river OR rivers OR sea OR “recreational space” OR “recreational spaces” OR “recreational area” OR “recreational areas” OR outdoor)

AND

TS=(inequality OR inequity OR inequities OR inequalities OR unequal OR "environmental justice" OR "environmental injustice") AND PY=(2010-2017)

Items selected manually: Results were additionally restricted by language (English) and document types (Article)

Scopus

ALL(disadvantaged OR disadvantage OR deprived OR social OR socio* OR vulnerable OR vulnerability OR psychosocial OR psycho-social OR socio-economic OR deprivation OR socio-demographic) AND TITLE-ABS-KEY(“green space” OR “green spaces” “open space” OR “open spaces” OR “natural space” OR “natural spaces” OR “green environment” OR “green environments” OR “green area” OR “green areas” OR greenery OR greenness OR “urban green” OR “public green” OR “neighbourhood green” OR “neighborhood green” OR “natural environment” OR “natural environments” OR park OR parks OR forest OR forests OR “urban park” OR “urban parks” OR “city park” OR “city parks” OR “park access” OR “public garden” OR “public gardens” OR “blue space” OR “blue spaces” OR “blue area” OR “blue areas” OR beach OR beaches OR lake OR lakes OR river OR rivers OR sea OR “recreational space” OR “recreational spaces” OR “recreational area” OR “recreational areas” OR outdoor) AND TITLE-ABS-KEY(inequality OR inequity OR inequities OR inequalities OR unequal OR "environmental justice" OR "environmental injustice") AND LANGUAGE(english) AND PUBYEAR > 2009 AND PUBYEAR < 2018 AND DOCTYPE(ar) AND NOT INDEX (medline)
